# Evaluation of a Novel Cisplatin Poloxamer Gel Formulation in the Treatment of Incompletely Excised Soft-Tissue Sarcomas: 42 Dogs

**DOI:** 10.3390/vetsci12030202

**Published:** 2025-02-27

**Authors:** Nicholas Lai, Veronika Langova, Penny Thomas, Sandra Nguyen, Johanna Todd, Joe Herbert, John Edward Blaxill

**Affiliations:** 1 Oncology Department, Small Animal Specialist Hospital, North Ryde, NSW 2113, Australiajherbert@umn.edu (J.H.);; 2 Internal Medicine Department, Western Australian Veterinary Emergency & Specialty, Success, WA 6164, Australia; 3 Veterinary Oncology Consultants, Central Coast, NSW 2259, Australia; 4 Small Animal Specialist Hospital, Prospect, NSW 2148, Australia; snguyen@sashvets.com (S.N.);; 5 Lewis Small Animal Hospital, College of Veterinary Medicine, University of Minnesota, St. Paul, MN 5518, USA

**Keywords:** soft-tissue sarcoma, canine, management, poloxamer, cisplatin, intralesional

## Abstract

Soft-tissue sarcomas are a heterogenous group of mesenchymal tumours that occur in dogs. Complete surgical excision is the ideal treatment for this tumour, but often, the location of the tumour makes this challenging, and the morbidity and cost of such a procedure may be prohibitive. This study describes the use of intralesional cisplatin in a novel poloxamer gel formulation, injected into the tumour bed as an adjuvant treatment to try and lower rates of local recurrence following incomplete and marginal excision. This formulation of cisplatin transiently solidifies at body temperature and exposes the tumour bed to high concentrations of this cytotoxic drug. An overall recurrence rate of 36% (15/42) was recorded in this cohort, with recurrence more likely to occur in tumours that had previously recurred and in larger (≥50 mm) tumours. Whilst this drug formulation is easy to administer and is well tolerated, subsequent use should be weighed against other adjuvant options. Other clinical utilisations of poloxamers in veterinary oncology should be explored.

## Simple Summary

Soft-tissue sarcomas are a group of skin tumours that are prone to local recurrence if not adequately surgically removed. This study describes a group of dogs in which a novel formulation of a chemotherapy agent (cisplatin) was injected into marginally excised tumour beds, in an attempt to reduce local recurrence rates. The results of this study help inform the future use of this drug and potentially lead to further investigative avenues for the use of these new compounds (poloxamers) in veterinary oncology.

## 1. Introduction

Soft-tissue sarcomas (STSs) are a heterogenous group of mesenchymal tumours that typically occur on the skin or subcutis of dogs [1]. Occasionally, more aggressive tumour subtypes such as haemangiosarcoma (HSA), osteosarcoma (OSA), and histiocytic sarcoma (HS) can be included in this umbrella term, but more often, these are considered separately given their distinct biological behaviours [2,3].

The local recurrence of the tumour following marginal excision is the main clinical challenge [4,5,6]. These tumours are often pseudoencapsulated, with a layer of compressed peritumoral connective tissue that can often harbour infiltrating tumour cells [7]. Oftentimes, obtaining wide surgical margins can be challenging or carry unacceptable morbidity due to the location of the tumour, particularly in the extremities [6,8].

Many adjuvant treatment options are available following marginal excision, including definitive or hypofractionated radiotherapy (RT) [9,10,11,12,13], electrochemotherapy [14,15], metronomic chemotherapy [16,17], and intralesional chemotherapy [18,19,20,21,22]. The infiltration of the local site with platinum drugs in various forms has previously been described, with varying degrees of toxicity. The high local concentrations of this drug with intralesional administration are thought to have the potential for cytotoxicity in the tumour bed and draining lymph nodes [20]. Generally, this mode of treatment does not seem to carry the same risk of nephrotoxicity that intravenous treatment does [19,23], possibly because delayed systemic absorption from the tumour site results in a lower maximal concentration of cisplatin in blood [20]. The intralesional treatment of a tumour bed is aimed at mopping up the microscopic disease left behind and is performed with a margin around a surgical scar to try and emulate surgical margins. The injection of chemotherapy into a gross tumour is typically not pursued with this disease due to intrinsic chemoresistance.

Poloxamers (thermoreversible hydrogels) for non-surgical drug delivery have constituted an active area of research. Poloxamer 407 is the most well described in the human literature [24] for intratumoural/peritumoural use, and its safety has also been demonstrated in dogs when used in an injectable/subcutaneous form [25,26]. A previous study in rodent models has identified it as a suitable agent to provide the local sustained release of platinum drugs whilst minimising systemic absorption [27]. It also has the advantage of being a compound already routinely utilised in topical applications.

The primary objective of this study was to describe the outcomes of dogs treated with a novel formulation of intralesional cisplatin in the adjuvant setting and to inform the future use of this drug.

## 2. Materials and Methods

Medical records for all dogs treated with the novel formulation of cisplatin as a poloxamer gel were identified with a search on practice software (Ezyvet version 41.8), and these records were examined. Patients were included if they had prior surgery to downstage disease, had a histopathological diagnosis of STS that was incompletely or marginally excised, and were treated with intralesional cisplatin (ILC) gel. Concurrent or prior treatment with NSAIDs and metronomic chemotherapy was allowed. Dogs treated with prior radiation were only allowed if their tumour had recurred in spite of the radiation.

Information collected from the medical record included age, breed, sex, weight, haematology/biochemistry blood tests, date of histopathological diagnosis, method of staging, date of ILC administration, date of last surgery prior to ILC administration, dose of drug used (mg/m^2^ and total mg), any local and systemic adverse effects in the period following administration, location, size (longest dimension on histopathology report), grade and mitotic count of tumour, histological margins, whether or not and when local recurrence was documented, date of death, and cause of death. Margins were determined to be infiltrated if there were tumour cells present at the margins and clean but close (CbCM) if tumour cells were present less than 5 mm from margins as defined by Monteiro et al., 2011 [1]. The location of the tumour was categorised as being in the extremity if it was at or distal to elbow/stifle joints as defined by Stefanello et al., 2008 [8], in the proximal limb (above elbow/stifle), or on the trunk (on the thoracic wall, rump, or perineum). Follow-up was performed by email to referring vets, and where records were not adequate to determine death or recurrence status, with phone calls to the owners.

### 2.1. Treatment with Intralesional Cisplatin

All dogs were placed under general anaesthesia for the instillation of chemotherapy at the local site. Peri-procedural intravenous fluid therapy was variably administered and/or recorded. The affected area was clipped and cleaned prior to instillation with a compounded formulation of cisplatin as a thermoreversible poloxamer gel (10 mg/mL; BOVA Aus, Caringbah, NSW, Australia). As this product warms, it increases in viscosity through the process of micellisation and gelation [24,28], forming a transient depot for the sustained release of cisplatin in the local area [27]. Specifically, Poloxamer 407, also known as Pluronic F127 [28] (20% *w*/*v* of poloxamer), was used to formulate a sterile aqueous solution of cisplatin suitable for subcutaneous injection.

**Figure 1:**
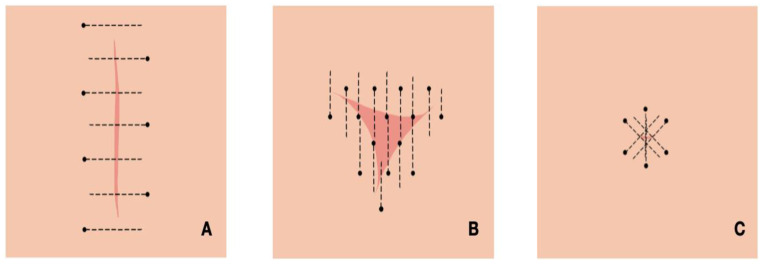
Illustrations of patterns of instillation of intralesional cisplatin into tumour beds of various geometries. Scar tissue is represented by a deeper pink on a beige background (normal skin). Sites of needle entry into the skin are demarcated by dots, with the subcutaneous needle tract represented by perforated lines. Where a linear scar was present, needle tracts perpendicular to the scar at intervals of 5 mm were used to cover the length of the scar (**A**). Where a treatment site was more square/triangular, a grid-like pattern of infiltration was used (**B**). For smaller treatment sites, a circumferential pattern of instillation centred around the tumour bed was used (**C**).

To try and ensure even and complete coverage of a tumour bed/scar, attempts to adhere to a standardised pattern of instillation depending on the geometry of the treatment site were made. Along a linear scar, ILC was administered on either side of the scar at 5 mm intervals (Figure 1A). If the tumour bed was less linear, then a grid pattern with 5 mm intervals between instillation sites (Figure 1B) was used. For a small scar/tumour bed, a circumferential pattern of instillation was employed (Figure 1C). With all of these techniques, the needle was directed into the subcutaneous space and withdrawn slowly while eluting small amounts of the cisplatin gel into the subcutis.

All dogs were treated once, with a maximal dose of 70 mg/m^2^ of cisplatin calculated. For larger dogs, a maximal dose of 70 mg was administered. The total dose was often limited by how much of the gel the subcutaneous space would accommodate. The median dose administered was 64 mg/m^2^ (23 mg–70 mg/m^2^), and treatment was administered at a median of 30 d (6–109 d) post-last surgical intervention. Two patients were treated with small amounts of residual disease present.

### 2.2. Metronomic Chemotherapy

Seven dogs also received metronomic chemotherapy after treatment with ILC. All dogs received daily oral doses of cyclophosphamide between 15 and 20 mg/m^2^, frusemide at 1 mg/kg, and a non-steroidal anti-inflammatory drug (NSAID). This was either meloxicam (0.1 mg/kg) or piroxicam (0.3 mg/kg).

### 2.3. Statistical Analysis

All analyses were performed using R statistical software (v4.4.1; R Core Team 2021; Vienna, Austria). Disease-free interval (DFI) was calculated from the date of ILC treatment to the date of documented progression. Survival time (ST) was calculated from the date of ILC treatment to the date of death/euthanasia for any cause. Median DFI and ST were estimated with the Kaplan–Meier method. Dogs were censored from progression-free analysis if their tumours had not recurred by the time of death and from survival analysis if they were still alive at the time of the final follow-up.

The stratification of patients into groups based on previously recorded prognostic criteria was also performed for the following variables:

1. Tumour margins (infiltrated or CbCM)
2. Tumour location (extremity or other)
3. Prior recurrence (yes or no)
4. Tumour grade (1, 2, or 3)
5. Tumour size (50 mm used as a cut-off as per Bray et al., 2014 [29] and Kuntz et al., 1997 [30])
6. Mitotic count (10 used as a cut-off as per McSporran, 2009 [31] and Kuntz et al., 1997 [30])

The log-rank test was used to assess differences in DFI/ST between groups. For all comparisons, a *p*-value of <0.05 was considered significant.

The distribution of a range of variables (e.g., age, sex, and weight) was assessed across groups with significantly different DFI/ST to identify any potentially confounding biases between the groups. Fisher’s exact, Mann–Whitney U, and t-tests were used to test for any significant differences between the groups for these variables.

## 3. Results

### 3.1. Patient and Tumour Characteristics

Forty-two (42) dogs treated at two locations (Small Animal Specialist Hospital North Ryde and Tuggerah) between the dates of October 2016 and December 2022 were retrospectively enrolled. Fifteen (15) were female neutered, and twenty-seven (27) were male neutered. Including crosses, there were seven Labradors; four Jack Russells and Rhodesian Ridgebacks; three Golden Retrievers, German Shepherds, and terriers; two Whippets, Kelpies, Beagles, Cavaliers, and Rottweilers; and one of each of the following breeds: Shiba Inu, Shih Tzu, Staffordshire Bull Terrier, Miniature Schnauzer, Poodle, Greyhound, Boxer, and a cross-breed. Age at diagnosis ranged from 4 to 14 years (median of 9 y). Patient weights ranged from 2.7 to 52.5 kg, with a median of 22.12 kg.

Most of the tumours were located on the extremity (30/42; 71%), with the rest located on the proximal limb (5/42; 12%) and trunk (7/42; 17%). Eleven (26%) tumours had recurred at least once prior to treatment with ILC. Staging was performed with a CT scan in most cases (25/42, 60%), with chest radiographs alone in nine cases (21%), and with chest radiographs and abdominal ultrasound in five cases (12%). Metastatic disease was not identified in any of these patients that were staged and went on to receive local treatment. Three patients were not staged.

Tumour size was recorded in 41 cases, and the longest dimension ranged from 4 to 105 mm, with a median of 35 mm. Grading was available in all cases, with 11/42 (26%) being grade I, 27/42 (65%) being grade II, and 4/42 (9%) being grade III. Margins were considered to be infiltrated in 21/42 (50%) cases, and CbCM in 21/42 (50%) cases. A summary of descriptive statistics is provided in Table 1.

### 3.2. Outcomes

**Figure 2:**
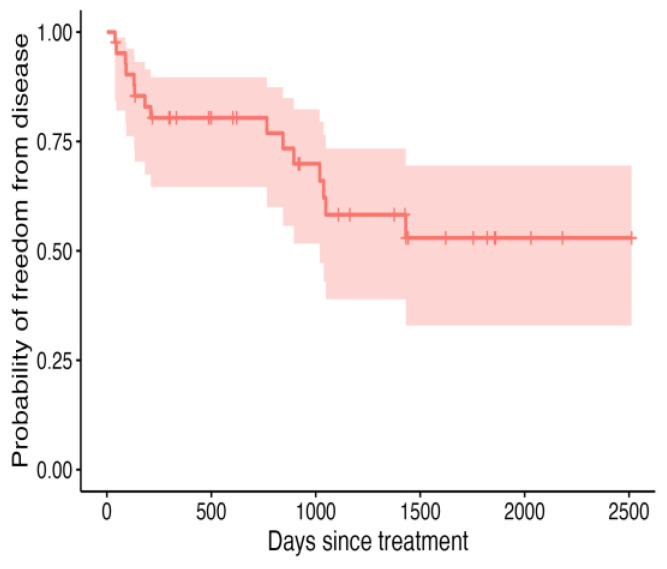
Kaplan–Meier curve depicting DFI for all 42 dogs treated with intralesional cisplatin, with shaded areas representing the 95% confidence intervals.

**Figure 3:**
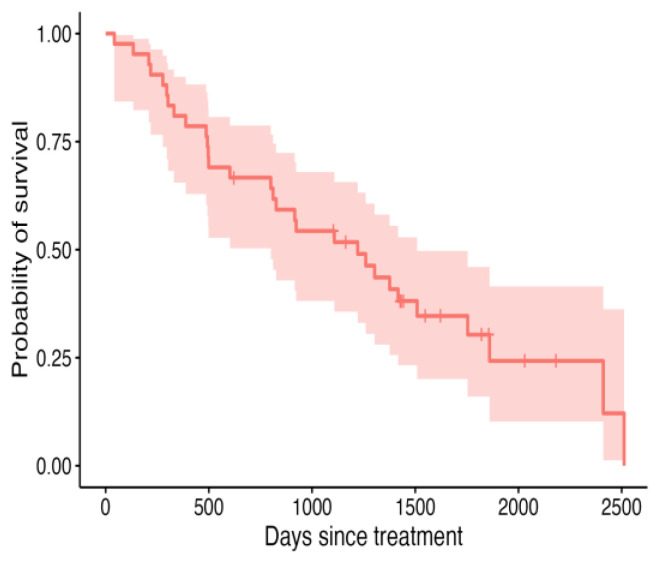
Kaplan–Meier curve depicting ST for all 42 dogs treated with intralesional cisplatin, with shaded areas representing the 95% confidence intervals.

An overall recurrence rate of 15/42 (36%) was recorded in this cohort of patients. In these 15 patients, the tumour recurred or progressed at a median of 210 d (19–1431 d). The median DFI was not reached (Figure 2), and the median ST was 1259 d (Figure 3). The median follow-up time for this population of dogs was 919.5 d (42–2512 d).

**Figure 4:**
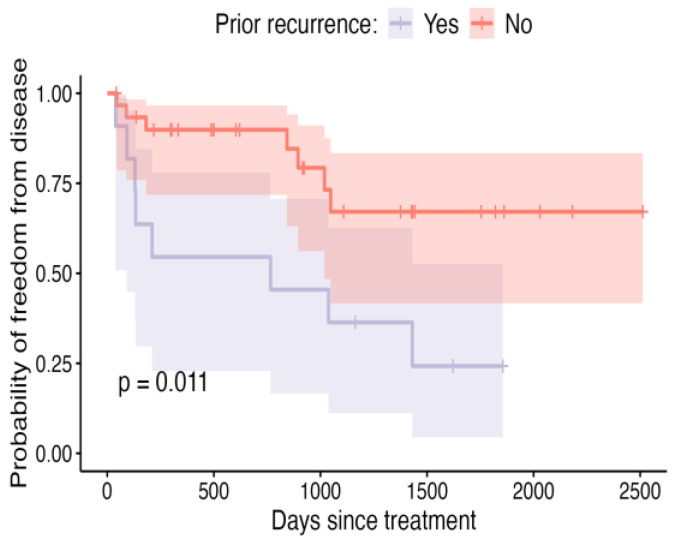
Kaplan–Meier curve depicting DFI for patients in which prior recurrence was recorded before ILC treatment, compared to patients in which ILC was administered after the first occurrence of the tumour, with shaded areas representing the 95% confidence intervals.

**Figure 5:**
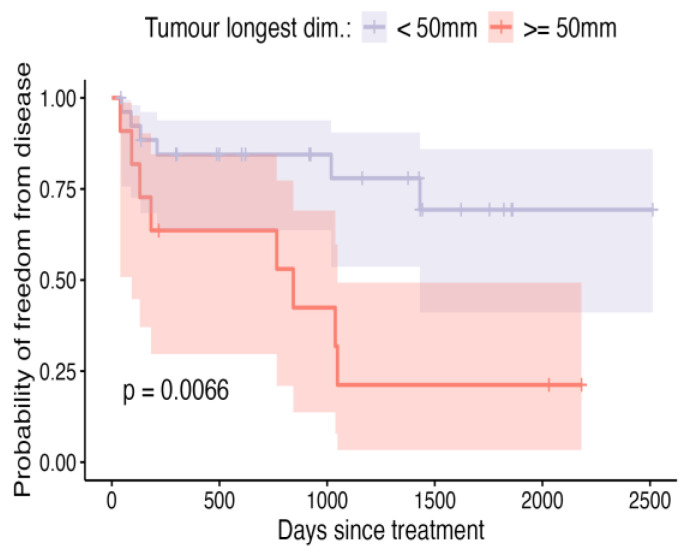
Kaplan–Meier curve depicting DFI for patients in which longest tumour dimension was ≥50 mm, compared to patients with tumours <50 mm, with shaded areas representing the 95% confidence intervals.

**Figure 6:**
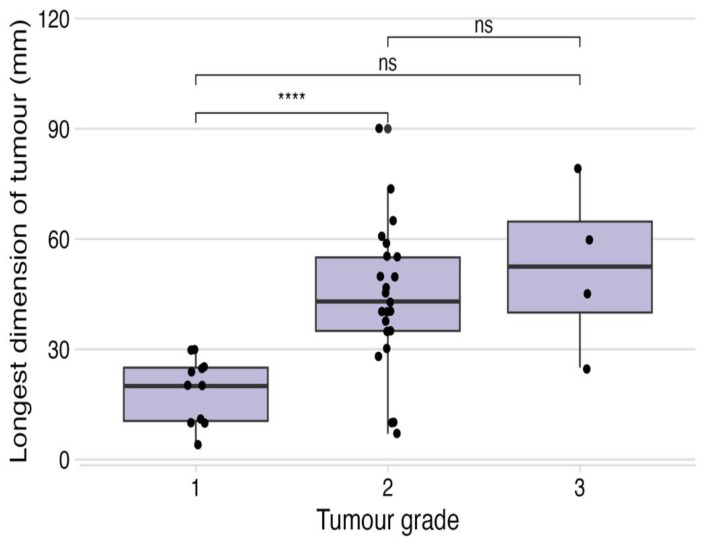
Box plot illustrating significant correlation between smaller tumour size and lower tumour grade in this cohort of patients (*t*-test, *p* < 0.01), with significance of grade 1 tumours and smaller size highlighted by (****), and non significance in size between grade 2/3 tumours demarcated by (ns).

Dogs with prior recurrence of their soft-tissue sarcomas were more likely to have them recur again after ILC, at a median of 900 d post-treatment (Figure 4; *p* = 0.01). Dogs with tumours where the longest dimension was ≥50 mm in size were also more likely to have their tumours recur at a median of 1036 d (Figure 5; *p* = 0.03), although, it is unclear how independent this variable is given the association of larger size and higher tumour grade in this cohort of dogs (Figure 6, *p* < 0.01).

**Figure 7:**
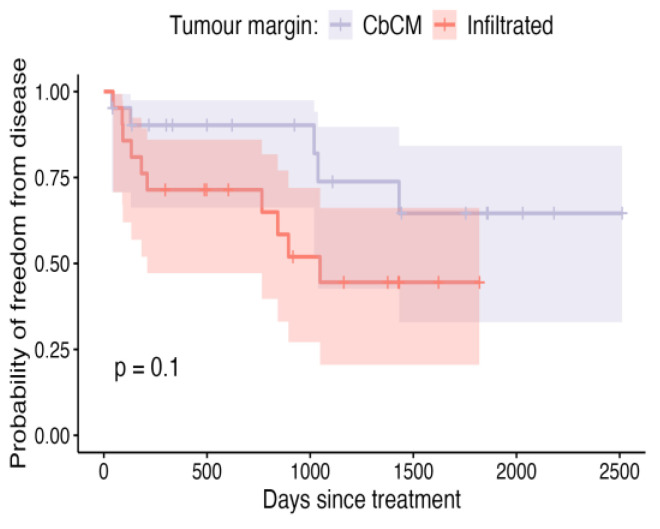
Kaplan–Meier curve depicting DFI for patients in which tumour margins were infiltrated, compared to clean but close margins (CbCM), with shaded areas representing the 95% confidence intervals.

**Figure 8:**
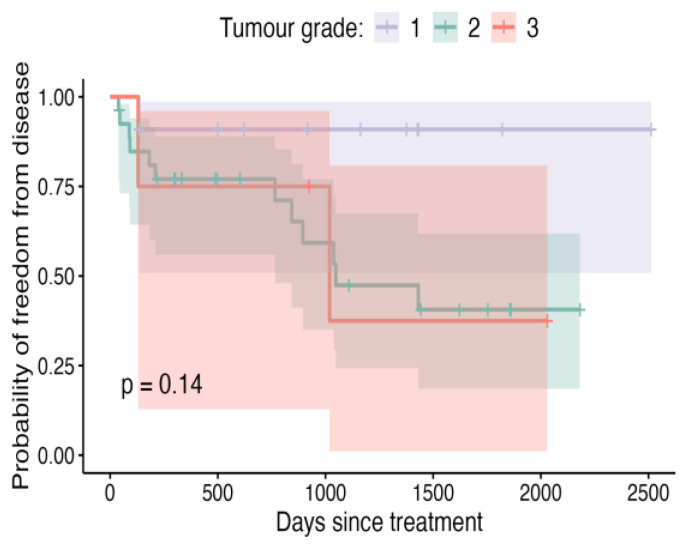
Kaplan–Meier curve depicting DFI for patients, stratified by tumour grade, with shaded areas representing the 95% confidence intervals.

**Figure 9:**
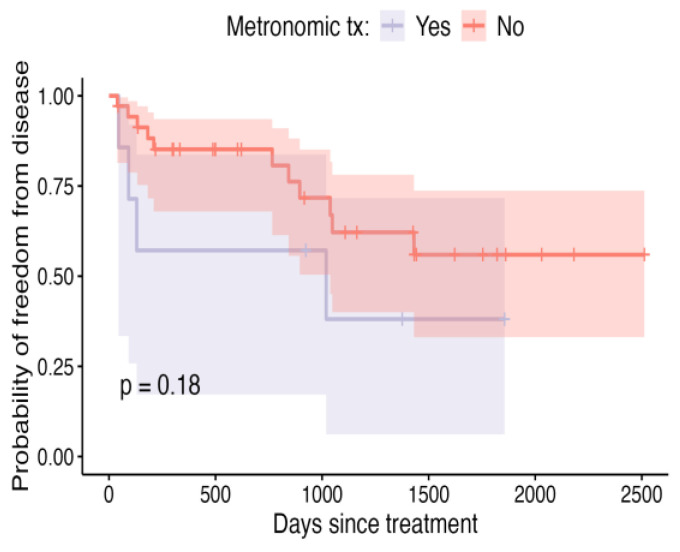
Kaplan–Meier curve depicting DFI for patients that received metronomic treatment (tx)—comprising cyclophosphamide, frusemide, and a non-steroidal anti-inflammatory drug following ILC treatment—compared to those that did not, with shaded areas representing the 95% confidence intervals.

Although not achieving conventional thresholds for statistical significance, neoplastic cells being present at the margins can be said to be predictive of recurrence compared to cases where there were clean but close (<5 mm) margins (Figure 7; *p* = 0.096). Also, grade 1 tumours were less likely to recur than their grade 2 or 3 counterparts (Figure 8, *p* = 0.14). Patients in which metronomic chemotherapy was used in conjunction with ILC experienced shorter, though not statistically different (Figure 9; *p* = 0.18), DFIs.

Tumour location (extremity vs. other) and mitotic count (<10, ≥10) were not prognostically significant in this patient population in terms of DFI (Figure S1a,b). Disease-free probabilities stratified by all examined variables are summarised in Table 2. None of the examined stratifications had clinically or statistically significant impacts on survival time (Figure S2a–h and Table S1).

Ten patients remain alive as of the time of the last follow-up. Of the 31 patients that died, 4 were recorded to have died due to local disease recurrence and progression, and 3 died of presumptive pulmonary metastasis (post-mortem histopathology of the lesions was not obtained). One patient was lost to follow-up. All of the others died due to causes unrelated to the cancer.

### 3.3. Toxicity

In total, 6/43 patients recorded local toxicity, with the most severe 3 of these recorded as grade 3 according to VCOG-CTCAE v2 [32]. These three patients experienced skin ulceration and had to have their treatment sites managed as open wounds. Two patients had transient oedema of the treatment site, and one patient was noted to be more tender on handling the limb the day after the procedure. One patient became acutely systemically unwell following the procedure and succumbed to acute kidney injury ~6 weeks after treatment. Signs of systemic unwellness (inappetence/vomiting) started the day after treatment, and bloodwork 4 days after treatment identified markedly increased creatinine. Bloodwork three weeks prior to treatment (at the time of surgery) showed only elevation in liver enzymes, but creatinine was within normal limits.

Of the 13 other patients in which creatinine was re-examined 2–4 weeks post-treatment, none had elevations concerning for acute kidney injury. The median change in creatinine amongst these 13 patients was an increase of 7 umol/L (range −20 umol/L to +26 umol/L).

## 4. Discussion

Intralesional cisplatin (ILC) as a poloxamer gel is appealing as an adjuvant treatment for marginally or incompletely excised canine STS in terms of being a once-off treatment that is relatively cost-efficient. It seems to be associated with fewer complications compared to previous ILC treatments [18,19,20,21], possibly owing to non-surgical administration techniques, after surgical wounds have had the chance to heal. Notably, the dose of the drug infiltrated locally by this formulation was also higher than the doses used in previous studies [18,19,20].

However, an overall recurrence rate of 36% in this study does call into question the utility of this agent in improving outcomes in incompletely or marginally excised STS. This recurrence rate is on the high end of what has previously been described for intralesional chemotherapy as an adjuvant treatment. It is comparable to patient populations that have previously been described [5], including those treated with other adjuvant modalities (Table 3).

Possible explanations for an increased rate of recurrence in this cohort would be the inclusion of histologically high-grade tumours and a high proportion of tumours (26%) that had already recurred following initial interventions. This would be consistent with the bias towards biologically more aggressive STSs in referral practice noted in a previous publication [26]. This cohort may be further skewed by the fact that some of these patients had tumours that recurred in spite of specialist intervention (surgery and in one patient, hypofractionated RT). As histopathology in these cases was performed by a variety of laboratories (and were not re-examined for this publication), it is possible that some included cases had tumours that behave more aggressively than STS (e.g., periarticular histolytic sarcoma). Interestingly, the tumours of the extremities in this cohort had a higher recurrence rate than a previously described cohort in which no adjuvant interventions were pursued [8].

The therapeutic benefit of the use of this formulation of intralesional cisplatin in use in recurrent tumours, as well as in tumours ≥50 mm in size is questionable. There are several possible reasons for larger tumour size being associated with higher rates of recurrence: a larger tumour bed being treated with a finite volume of cisplatin solution may predispose to geographic misses, and in addition, larger tumours in previous publications have been posited to display more biologically aggressive behaviours [29,30]. No tumour ≥50 mm in this cohort was grade 1, which makes it challenging to elicit whether the increased risk of recurrence is due to tumour size or higher tumour grade.

No conclusions could be made regarding the use of metronomic chemotherapy from this cohort of dogs, given the small number of treated patients and the lack of randomisation in terms of which patients were given treatment. It seems likely, given the patients treated with metronomic chemotherapy seemed to fare worse than their non-treated counterparts, that patients with tumours with negative prognostic indicators were chosen to be treated. Given the possible systemic toxicity in one patient, peri-procedural intravenous fluids should be strongly considered where there is no contraindication (e.g., severe cardiac disease). More stringent renal screening, including urinalysis and creatinine measurements immediately before and two weeks after drug administration, will be needed to determine if the local infiltration of cisplatin has a significant risk for nephrotoxicity.

Poloxamer 407 as a vehicle for the subcutaneous delivery of chemotherapy drugs seems to be a promising avenue of study for tumours in which the recurrence of local disease is a significant concern. Based on this cohort of dogs, this drug formulation seems to be very well tolerated, with the only severe adverse effect identified being more likely related to the cisplatin element of the drug, or even possibly to anaesthetic-related complications.

The major limitation of this study was the lack of a contemporaneous control group. Also, the histological subtype of each of these tumours was not able to be determined—this may be significant as perivascular wall tumours have previously been shown to recur less frequently [5]. Margins < 5 mm were categorised as incomplete for the purpose of this study, recognising that this is an area lacking consensus [34] and that some of the patients that were treated would have what others would consider complete margins.

The retrospective nature of the study also meant that there was variation in recheck schedules and also in the completeness of records available for analysis. Gross characteristics of the tumour that have previously been described as prognostically significant, such as whether or not they were fixed/invasive or mobile [35], and their size in situ were often not recorded accurately at the time of surgical removal. The most consistent reporting on the size of the tumour, which was utilised for this publication, was in the histopathology reports, which describe tumour dimensions after formalin fixation. As some patients returned to their referring practices for follow-up examination following treatment, toxicities may be under-reported as mild toxicity that did not require active management was unlikely to be recorded. The other limitation of this study is the small sample size, which made conducting a multivariate Cox regression analysis less useful in trying to work out which of the prognostic factors are truly significant [36].

Further applications of this drug formulation in other species and oncologic diseases can be considered. Future studies in this area are warranted to characterise the release profile of cisplatin from the poloxamer gel after injection, as well as to examine the spatial distribution and dispersal of the poloxamer gel (e.g., with fluorescence dye conjugated poloxamer) after injection. Cisplatin is still commonly used for equine sarcoids [37], and leakage of the drug from the infiltration sites may contribute to treatment failure in addition to being an occupational health and safety hazard for personnel. The viscosity of this drug formulation and its properties (turning to a semi-solid state in vivo) may make it less likely to leak or be aerosolised and may be more ideal for this application. Similar poloxamer/hydrogels can also be considered in the delivery of anti-metabolite agents like cytosine arabinoside or gemcitabine, which typically require a constant rate of infusion via an intravenous delivery system, although pharmacokinetic studies will need to be performed.

## Figures and Tables

**Table 1:** Summary of descriptive statistics for patient population of 42 dogs treated with intralesional cisplatin following incomplete excision of soft-tissue sarcoma.

Variable	Category	N	%
Breed	Labrador	7	17
	Jack Russell Terrier	4	9.5
	Rhodesian Ridgeback	4	9.5
	Golden Retriever	3	7
	German Shepherd	3	7
	Other	21	50
Staging	CT Chest/Abdomen	25	60
	Chest Radiographs +/− AUS	14	33
	Not Staged	3	7
Anatomic Location	Extremity	30	71
	Other	12	29
Margins	Infiltrated	21	50
	CbCM	21	50
Grade	1	11	26
	2	27	64
	3	4	9
Previous recurrence	Yes	11	26
	No	31	74
Metronomic chemo-therapy	Yes	7	17
	No	35	83

**Table 2:** Summary of disease-free probabilities tracked over 1, 3, and 5 years, with significant values highlighted in bold and demarcated with (**). Where progression was noted in less than 50% of the population, median DFI could not be calculated, and is recorded as NA.

Variable	Category	1 Year	3 Years	5 Years	Median DFI (d)	*p*-Value
All dogs		0.80 (0.65–0.90)	0.58 (0.39–0.73)	0.53 (0.33–0.74)	NA	
Prior recurrence	Yes	0.55 (0.23–0.78)	0.36 (0.11–0.63)	0.24 (0.04–0.53)	767	0.01 **
	No	0.90 (0.72–0.97)	0.67 (0.42–0.83)	0.67 (0.42–0.83)	NA	
Tumour margin	Infiltrated	0.71 (0.47–0.86)	0.45 (0.21–0.66)	0.45 (0.21–0.66)	1048	0.1
	CbCM	0.90 (0.66–0.97)	0.74 (0.43–0.90)	0.65 (0.33–0.84)	NA	
Tumour location	Extremity	0.83 (0.63–0.92)	0.55 (0.32–0.72)	0.55 (0.32–0.72)	NA	0.89
	Other	0.75 (0.41–0.91)	0.75 (0.41–0.91)	0.5 (0.09–0.82)	1431	
Tumour grade	1	0.91 (0.51–0.99)	0.91 (0.51–0.99)	0.91 (0.51–0.99)	NA	0.14
	2	0.77 (0.56–0.89)	0.47 (0.24–0.67)	0.41 (0.18–0.62)	1048	
	3	0.75 (0.13–0.96)	0.38 (0.01–0.81)	0.38 (0.01–0.81)	1018	
Tumour dimension	<50 mm	0.84 (0.64–0.94)	0.78 (0.54–0.91)	0.69 (0.41–0.86)	NA	0.01 **
	≥50 mm	0.64 (0.30–0.85)	0.21 (0.03–0.49)	0.21 (0.03–0.49)	843	
Mitotic count	<10	0.80 (0.58–0.91)	0.62 (0.37–0.79)	0.62 (0.37–0.79)	NA	0.44
	≥10	0.80 (0.5–0.93)	0.51 (0.20–0.75)	0.41 (0.13–0.67)	1431	
Metronomic chemotherapy	Yes	0.57 (0.17–0.84)	0.38 (0.06–0.72)	0.38 (0.06–0.72)	1018	0.18
	No	0.85 (0.68–0.94)	0.62 (0.40–0.78)	0.56 (0.33–0.74)	NA	

**Table 3:** Summary of local recurrence rates recorded with various adjuvant treatment modalities following surgical excision of canine STS.

Adjuvant Treatment	Recurrence Rate (%)	References
Intralesional chemotherapy	17–33	[18,19,20,21,22]
Electrochemotherapy	14–24	[14,15]
Hypofractionated RT	18–21	[9,10]
Hyperfractionated RT	17–31	[11,12,13]
None	11–44	[8,29,31,33]

## Data Availability

The authors confirm that the data supporting the findings of this study are available within the article [and/or] its Supplementary Materials.

## Supplementary Materials

The following supporting information can be downloaded at https://www.mdpi.com/article/10.3390/vetsci12030202/s1, Figure S1a: KM curve depicting DFI for patients in which mitotic count <10, compared to patients in which this was ≥10; Figure S1b: KM curve depicting DFI for patients in which tumours were located in the extremities, compared to in other locations (proximal limb, trunk); Figure S2a: KM curve depicting ST for patients in which prior recurrence of the tumour was noted before ILC treatment, compared to patients in which ILC was administered after first occurrence of the tumour; Figure S2b: KM curve depicting ST for patients in which tumour margins were infiltrated, compared to clean but close margins (CbCM); Figure S2c: KM curve depicting ST for patients in which tumours were located in the extremities, compared to in other locations (proximal limb, trunk); Figure S2d: KM curve depicting ST for patients, stratified by tumour grade; Figure S2e: KM curve depicting ST for patients in which longest tumour dimension was ≥50mm, compared to patients with tumours <50 mm; Figure S2f: KM curve depicting ST for patients in which mitotic count <10, compared to patients in which this was ≥10; Figure S2g: KM curve depicting ST for patients that received metronomic chemotherapy after ILC treatment, compared to those that did not; Figure S2h: KM curve depicting ST for patients in which local recurrence was noted following ILC treatment, compared to those that remained disease free; Table S1: Summary of survival probabilities tracked over 1, 3 and 5 years.
