# Supplementary material for: Evaluation of a Novel Cisplatin Poloxamer Gel Formulation in the Treatment of Incompletely Excised Soft-Tissue Sarcomas: 42 Dogs
Source: Vet Sci. 2025 Feb 27;12(3):202. doi: 10.3390/vetsci12030202 (PMC11946595; doi:10.3390/vetsci12030202)
Supplement: Supplementary file 1 [file vetsci-12-00202-s001.zip › vetsci-3323016-supplementary.pdf]

Supplementary Material

Supplementary Figures S1

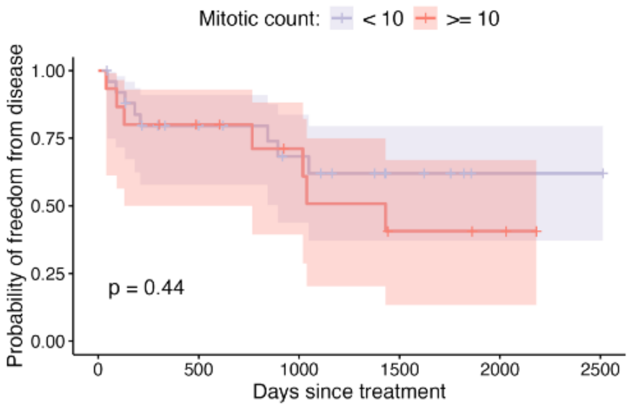

Figure S1a KM curve depicting DFI for patients in which mitotic count <10, compared to patients in which this was ≥10.

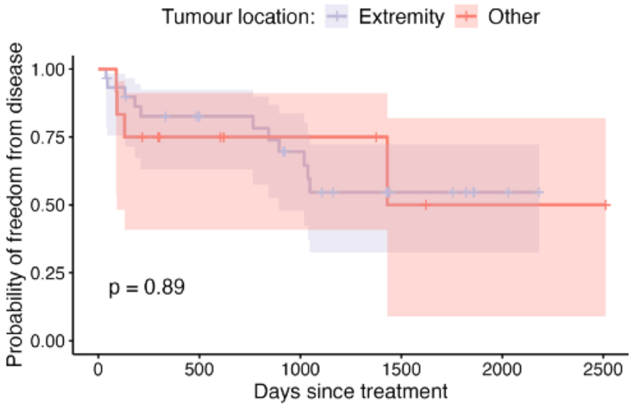

Figure S1b KM curve depicting DFI for patients in which tumours were located in the extremities, compared to in other locations (proximal limb, trunk).

Supplementary Figures S2

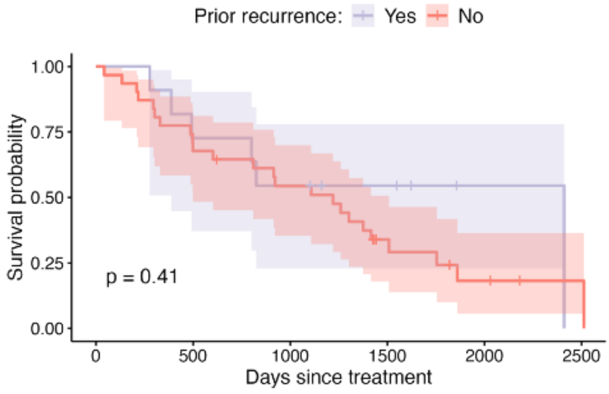

Figure S2a KM curve depicting ST for patients in which prior recurrence of the tumour was noted before ILC treatment, compared to patients in which ILC was administered after first occurrence of the tumour.

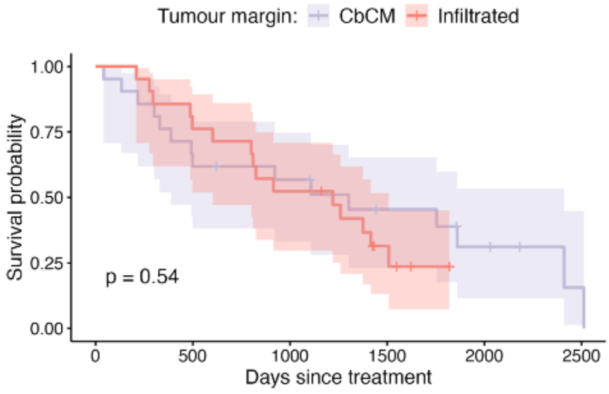

Figure S2b KM curve depicting ST for patients in which tumour margins were infiltrated, compared to clean but close margins (CbCM).

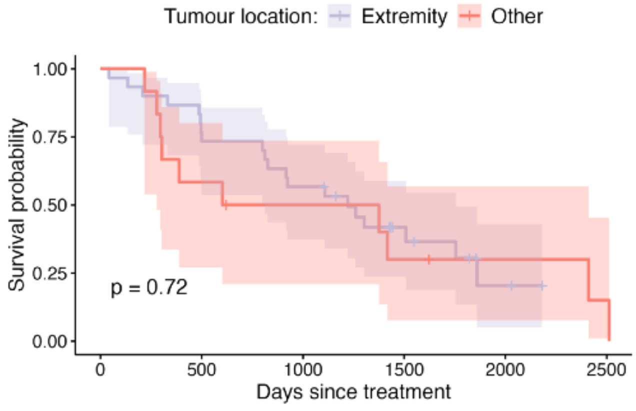

Figure S2c KM curve depicting ST for patients in which tumours were located in the extremities, compared to in other locations (proximal limb, trunk).

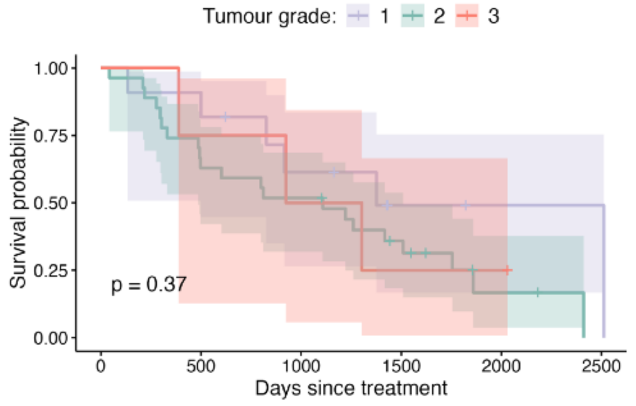

Figure S2d KM curve depicting ST for patients, stratified by tumour grade

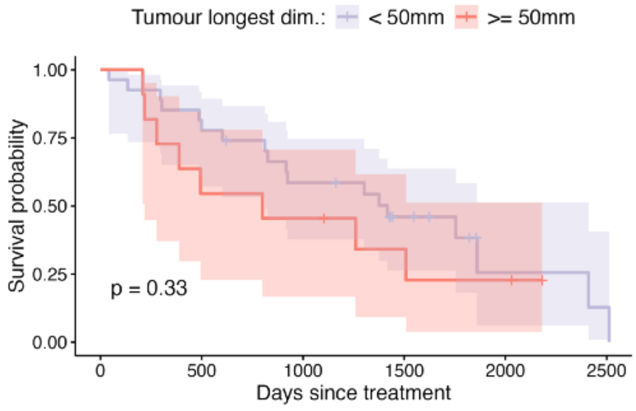

Figure S2e KM curve depicting ST for patients in which longest tumour dimension was ≥50mm, compared to patients with tumours <50mm.

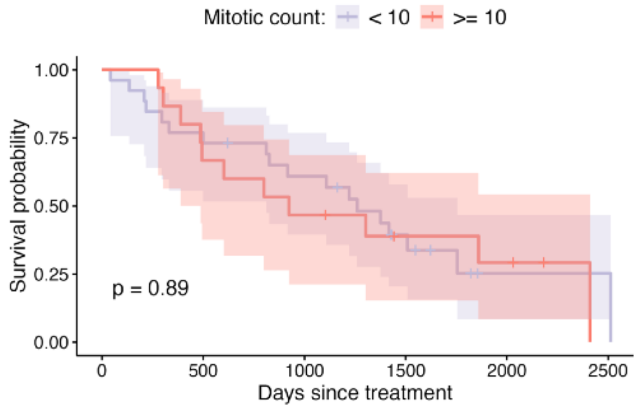

Figure S2f KM curve depicting ST for patients in which mitotic count <10, compared to patients in which this was ≥10.

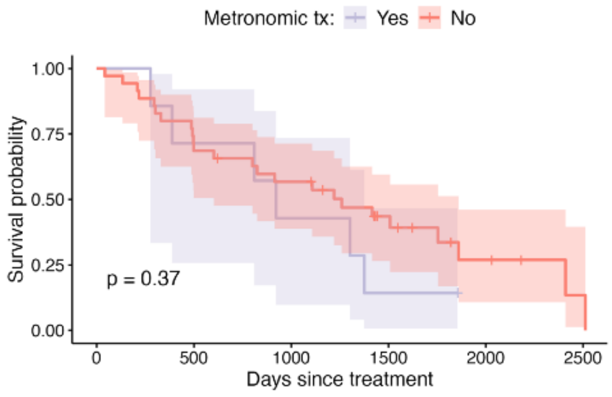

Figure S2g KM curve depicting ST for patients that received metronomic chemotherapy after ILC treatment, compared to those that did not.

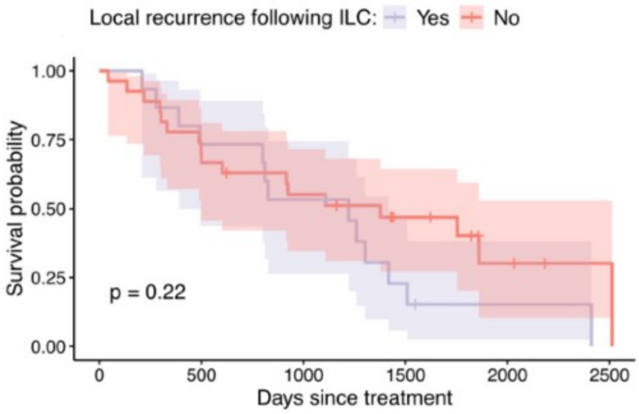

Figure S2h KM curve depicting ST for patients in which local recurrence was noted following ILC treatment, compared to those that remained disease free.

Supplementary Table S1

Table S1 Summary of survival probabilities tracked over 1, 3 and 5 years.

Variable	Category	1 year	3 years	5 years	Median ST (d)	P-value
All dogs		0.81 (0.66-0.90)	0.54 (0.38-0.68)	0.30 (0.16-0.46)	1223	
Prior recurrence	Yes	0.91 (0.51-0.99)	0.55 (0.23-0.78)	0.55 (0.23-0.78)	2413	0.41
	No	0.77 (0.58-0.89)	0.54 (0.35-0.70)	0.24 (0.10-0.42)	1223	
Tumour margin	Infiltrated	0.86 (0.62-0.95)	0.52 (0.30-0.71)	0.24 (0.07-0.45)	1223	0.54
	CbCM	0.76 (0.52-0.89)	0.57 (0.33-0.75)	0.39 (0.18-0.60)	1303	
Tumour location	Extremity	0.87 (0.68-0.95)	0.57 (0.37-0.72)	0.30 (0.13-0.49)	1223	0.72
	Other	0.67 (0.34-0.86)	0.5 (0.21-0.74)	0.3 (0.08-0.57)	989	
Tumour grade	1	0.91 (0.51-0.99)	0.61 (0.27-0.84)	0.49 (0.17-0.75)	1376	0.37
	2	0.74 (0.53-0.87)	0.52 (0.32-0.69)	0.25 (0.10-0.44)	1110	
	3	1.0 (1.0-1.0)	0.5 (0.06-0.84)	0.25 (0.01-0.67)	1113	
Tumour dimension	<50mm	0.85 (0.65-0.94)	0.58 (0.38-0.75)	0.38 (0.18-0.58)	1416	0.33
	≥50mm	0.73 (0.37-0.90)	0.45 (0.17-0.71)	0.23 (0.04-0.51)	799	
Mitotic count	<10	0.77 (0.56-0.89)	0.61 (0.39-0.77)	0.25 (0.08-0.47)	1259	0.89
	≥10	0.87 (0.56-0.96)	0.47 (0.21-0.69)	0.39 (0.15-0.62)	923	
Metronomic chemotherapy	Yes	0.86 (0.33-0.98)	0.43 (0.10-0.73)	0.14 (0.01-0.46)	923	0.37
	No	0.80 (0.63-0.90)	0.57 (0.39-0.71)	0.34 (0.17-0.51)	1259	
Local recurrence following ILC	Yes	0.87 (0.56-0.96)	0.53 (0.26-0.74)	0.15 (0.03-0.38)	1223	0.22
	No	0.78 (0.57-0.89)	0.55 (0.35-0.72)	0.4 (0.20-0.59)	1376	
